# Caring for Hospitalized Patients with Substance Use Disorders: An Interprofessional Needs Assessment Survey

**DOI:** 10.1016/j.ajmo.2025.100116

**Published:** 2025-08-20

**Authors:** M. Holliday Davis, Judy Chertok, Shoshana Aronowitz, Rachel French, Jeanmarie Perrone, Ashish P Thakrar, Samantha Huo, Jacqueline Deanna Wilson, Nia Bhadra-Heintz, Lilah Lesniak, Aidan Hecker, Jessica Tolbert, Margaret Lowenstein

**Affiliations:** a Department of Medicine, Perelman School of Medicine at the University of Pennsylvania, Philadelphia, Pa; b University of Pennsylvania Center for Addiction Medicine and Policy, Philadelphia, Pa; c University of Pennsylvania School of Nursing, Philadelphia, Pa; d Department of Family Medicine and Community Health, Perelman School of Medicine at the University of Pennsylvania, Philadelphia, Pa; e Leonard Davis Institute of Health Economics, Philadelphia, Pa; f Department of Emergency Medicine, Perelman School of Medicine at the University of Pennsylvania, Philadelphia, Pa; g Department of Obstetrics and Gynecology, Perelman School of Medicine at the University of Pennsylvania, Philadelphia, Pa; h University of Pennsylvania School of Arts and Sciences

**Keywords:** Addiction, Addiction consult service, Clinician burnout, Harm reduction, Hospital-based addiction care, Inpatient care, Interprofessional teams, Mixed methods, People who use drugs, Qualitative, Substance use disorder, Survey

## Abstract

**Background:**

Hospitalizations among people who use drugs (PWUD) are increasing, and addiction consult services (ACS) are an emerging best practice for improving care.

**Methods:**

We conducted a web-based needs assessment survey of physicians, advanced practice providers (APP), and nurses at a Philadelphia academic hospital in March 2023 before implementing an ACS. We assessed knowledge gaps, barriers to care, and perceived service needs.

**Results:**

Of 472 clinicians surveyed, 236 responded (50% response rate). Participants felt most prepared to assess withdrawal and diagnose or recognize substance use disorders (SUDs) but lacked confidence in care linkage and harm reduction. Reported barriers included patient social needs, resource availability, and lack of expert consultation.

**Conclusions:**

While most participants agreed that SUDs are treatable, many reported compromised patient care due to inadequate support as well as burnout associated with caring for PWUD. Future work should examine whether ACSs address the perceived barriers to care for hospitalized PWUD while supporting clinicians.

## Graphical Abstract

**Figure:**
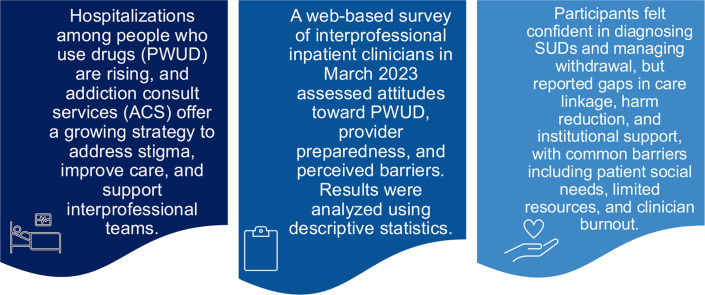

## Introduction

Although overdose mortality is declining, serious morbidity is increasing among people who use drugs (PWUD), contributing to rising hospital admissions, readmissions, and overall hospital days.^1^^,^^2^ Hospitalization can be an important moment to engage people in addiction treatment and other services, however PWUD continue to face barriers to care in hospital settings, including knowledge gaps among providers, inadequate linkage after discharge, and stigma.3., 4., 5., 6.

Addiction consult services (ACS) are a growing strategy to improve patient outcomes and support hospital teams providing addiction care.^7^^,^^8^ Despite their increasing adoption, there is limited guidance on how to tailor ACS models to the specific needs of different hospital settings and clinical teams. Baseline data on clinician knowledge, attitudes, and perceived barriers to addiction care are critical to inform the design and successful implementation of these services. Prior to the implementation of an ACS at an academic hospital in Philadelphia, we conducted a needs assessment with hospital clinicians. Our aim was to characterize attitudes, perceived preparedness to care for patients with SUD, current services, and perceived barriers to addiction care.

## Methods

### Study Design and Population

We conducted a web-based, cross-sectional survey of inpatient clinicians prior to the implementation of an ACS at a single hospital in Philadelphia, PA in March 2023. The study was approved by the University of Pennsylvania Institutional Review Board. The urban, academic hospital was implementing an ACS the following month and experiences a high volume of admissions for patients with substance use disorders (SUD).

### Survey Content and Administration

The survey assessed 3 domains: provider attitudes towards PWUD, self-rated preparation for aspects of SUD care, and perceived barriers to providing these services (see Appendix A for full instrument). Questions were adapted from prior work conducted in hospitals and other settings.9., 10., 11. Attitudes were measured using a 5-point Likert scale (Strongly disagree, Somewhat disagree, Neutral, Somewhat agree, Strongly agree), and comfort with SUD care was assessed using a separate 5-point Likert scale ranging from Very unprepared to Very prepared. Barriers were assessed using a 3-point scale (Not a barrier, Somewhat of a barrier, Significant barrier). We also collected demographic data, including professional role, years of experience, and optional self-disclosure of having family or friend with SUD. Participants self-identified their gender, race and ethnicity using fixed-choice categories consistent with U.S. Census Bureau classifications, with an option to select ‘Other’ and specify. Race and ethnicity were collected as separate items. These variables were assessed to describe differences in perspectives across demographic groups. Responses coded as ‘Other’ were reviewed and included in the appropriate category or retained as separate, as applicable. The survey instrument was piloted with a group of inpatient clinicians to ensure clarity, relevance, and applicability.

We invited attending physicians, resident physicians, advanced practice providers (APPs) and nurses from hospital medical and surgical floors to participate via email, with up to 3 reminders. The survey was self-administered by respondents using the REDCap secure web platform. Participants received a $10 incentive for completing the survey.

### Data Analysis

We used descriptive statistics to characterize the sample and responses. Likert-scale responses for preparedness and attitudes were dichotomized for analysis (eg, combining “somewhat prepared” and “very prepared” into a single “prepared” category). We used chi-squared (χ²) tests to assess differences in proportions across provider groups. Analyses were conducted using Stata.

## Results

### Respondent Characteristics

There were 236 respondents out of a sample of 472 (response rate of 50%). This included 128 resident physicians (54%), 58 nurses (25%), 36 attending physicians (15%), and 14 advanced practice providers (APP) (6%) (Table 1). Respondents were mostly female (72%), followed by male (27%), nonbinary (1%), and other unspecified (1%). Participants were predominantly White (58%), followed by Asian (19%), Black (14%), and other/unspecified (9%). Total 92% of participants were Non-Hispanic/Latine. Total 28% of respondents reported having a close friend or family member with SUD.

**Table 1:** Demographics.

Characteristic	Total, n (%)	Attending Physicians, n (%)	APPs, n (%)	Resident Physicians, n (%)	Nurses, n (%)	p-Value
Total	236 (100%)	36 (15.3%)	14 (6%)	128 (54%)	58 (25%)	
Gender						
Female	169 (72%)	25 (70%)	13 (93%)	81 (63%)	50 (86%)	.004
Male	63 (27%)	11 (31%)	1 (7%)	46 (36%)	5 (9%)	
Nonbinary	2 (1%)	0 (0%)	0 (0%)	1 (1%)	1 (2%)	
Other	2 (1%)	0 (0%)	0 (0%)	1 (1%)	3 (5%)	
Race						
Black	33 (14%)	0 (0%)	2 (14%)	12 (9%)	19 (33%)	< .001
White	137 (58%)	20 (55%)	10 (71%)	86 (67%)	21 (36%)	
Asian	44 (19%)	12 (33%)	1 (7%)	22 (17%)	9 (16%)	
Native American/American Indian	1 (1%)	0 (0%)	0 (0%)	0 (0%)	1 (1%)	
Other	21 (9%)	4 (11%)	1 (7%)	8 (6%)	9 (16%)	
Ethnicity						
Hispanic/Latine	20 (8%)	1 (3%)	0 (0%)	15 (12%)	4 (7%)	.193
Non-Hispanic/Latine	216 (92%)	35 (97%)	14 (100%)	113 (88%)	54 (93%)	
Close Friend or Family with SUD						
Yes	66 (28%)	3 (8%)	5 (36%)	36 (28%)	22 (38%)	.023
No	151 (64%)	30 (83%)	7 (50%)	85 (66%)	29 (50%)	
Did not Answer	19 (8%)	3 (8%)	2 (14%)	7 (5%)	7 (12%)	

### Attitudes Regarding SUD Care

Most participants (81%) believed that patients with SUD are more challenging to care for than those without SUD, with APP (100%) and attending physicians (83%) reporting the highest agreement (Table 2). While 91% of respondents agreed SUD is treatable, this belief varied, with resident physicians expressing the highest agreement (96%) and nurses the lowest (78%). Concerns about time constraints were common, with 51% of respondents reported feeling they lacked sufficient time to care for patients with SUD. Concerns about patient interactions were also common, with 35% of respondents worrying about enabling addiction and 40% feeling manipulated by patients with SUD.

**Table 2:** Attitudes, Knowledge, and Barriers to SUD Care by Group.

Attitudes, Proportion somewhat or strongly agreeing	Total, n (%)	Resident Physicians, n (%)	Nurses, n (%)	Attending Physicians, n (%)	APP, n (%)	p-Value
Patients with SUD are more challenging to care for than patients w/o addiction	190 (81%)	103 (80%)	43 (74%)	30 (83%)	14 (100%)	.167
SUD is treatable	214 (91%)	123 (96%)	45 (78%)	33 (92%)	13 (93%)	<.001
I don't have enough time to care for patients with SUD	120 (51%)	68 (53%)	25 (43%)	19 (52%)	8 (57%)	.586
I worry about worsening or enabling addiction in my patients	83 (35%)	36 (28%)	28 (48%)	11 (31%)	8 (57%)	.015
I feel manipulated by my patients with SUD	94 (40%)	44 (34%)	27 (47%)	15 (42%)	8 (57%)	.214
I feel unsupported in caring for patients with SUD	97 (41%)	53 (41%)	22 (38%)	15 (42%)	7 (50%)	.871
Caring for patients with SUD contributes to burnout for me	129 (55%)	67 (52%)	34 (59%)	17 (47%)	11 (79%)	.197
There have been times I have had to provide SUD care I did not feel qualified caring for	128 (54%)	72 (56%)	23 (40%)	23 (64%)	10 (71%)	.042
I have witnessed compromised care due to lack of support for addiction treatment	184 (78%)	107 (84%)	34 (59%)	31 (86%)	12 (86%)	.001
I have felt distress when witnessing stigmatizing treatment of patients w/ SUD	175 (74%)	108 (84%)	32 (55%)	26 (82%)	9 (64%)	.001
Caring for patients with SUD is one of the most difficult parts of my job	96 (41%)	45 (35%)	29 (50%)	16 (44%)	6 (42%)	.267


Comfort with SUD care, Proportion reporting somewhat or very comfortable	Total, n (%)	Resident Physicians, n (%)	Nurses, n (%)	Attending Physicians, n (%)	APP, n (%)	p-Value
Assess for alcohol WD	212 (89%)	116 (91%)	48 (83%)	34 (94%)	14 (100%)	.128
Assess for opioid WD	217 (92%)	120 (94%)	49 (84%)	34 (94%)	14 (100%)	.091
Diagnose/Recognize SUD	208 (88%)	117 (91%)	47 (81%)	32 (89%)	12 (86%)	.240
Discuss MOUDs*Nurses excluded	139 (78%)	102 (80%)	N/A	31 (86%)	6 (43%)	.003
Initiate/Administer methadone in the hospital	165 (70%)	90 (70%)	48 (83%)	21 (58%)	6 (43%)	.008
Initiate/Administer buprenorphine in the hospital	168 (71%)	91 (71%)	45 (78%)	28 (77%)	4 (29%)	.003
Manage/Address acute pain in patients with SUD	168 (71%)	96 (75%)	40 (69%)	25 (69%)	7 (50%)	.244
Discuss MAUDs (Nurses excluded)	87 (49%)	61 (48%)	N/A	24 (67%)	2 (14%)	.003
Provide wound care for wounds related to IV drug use (Physicians and APPs excluded)	N/A	N/A	47 (81%)	N/A	N/A	N/A
Counsel patients on overdose reversal with naloxone	169 (72%)	102 (80%)	32 (55%)	29 (81%)	6 (43%)	<.001
Address in-hospital drug use	89 (38%)	48 (38%)	22 (38%)	13 (36%)	6 (43%)	.977
Counsel patients on safer ways to drink alcohol	83 (35%)	42 (33%)	21 (36%)	15 (42%)	5 (36%)	799
Counsel patients on safer drug use practices	97 (41%)	54 (42%)	24 (41%)	16 (44%)	3 (21%)	.481
Determine an appropriate LOC for SUD (Nurses excluded)	76 (43%)	56 (44%)	N/A	15 (42%)	5 (36%)	.838
Connect patients to outpatient SUD treatment (Nurses excluded)	55 (31%)	38 (30%)	N/A	13 (36%)	4 (29%)	.748


Barriers	Total, n (%)	Resident Physicians, n (%)	Nurses, n (%)	Attending Physicians, n (%)	APP, n (%)	p-Value
Patient social barriers	199 (84%)	117 (91%)	38 (66%)	32 (89%)	12 (86%)	<.001
Availability of resources after discharge	160 (68%)	99 (77%)	20 (34%)	29 (81%)	12 (86%)	<.001
Access to expert consultation	131 (56%)	77 (60%)	22 (38%)	22 (61%)	10 (71%)	.016
Access to harm reduction resources	126 (53%)	73 (57%)	24 (41%)	21 (58%)	8 (57%)	.214
Access to social work support	112 (47%)	63 (49%)	16 (28%)	25 (69%)	8 (57%)	.001
Access to peer support	86 (36%)	41 (32%)	19 (33%)	18 (50%)	8 (57%)	.076
Knowledge about SUD tx	46 (19%)	25 (20%)	7 (12%)	8 (22%)	6 (43%)	.069
Patient interest in treatment	109 (46%)	55 (43%)	30 (52%)	13 (36%)	11 (79%)	.035
Guidelines for managing OUD	46 (19%)	22 (17%)	9 (16%)	10 (28%)	5 (36%)	.176
Knowledge about pain management in patients with SUD	75 (32%)	43 (34%)	13 (22%)	14 (39%)	5 (36%)	.323

Participants also reported challenges in caring for patients with SUD. Overall, 41% felt unsupported in providing SUD care and 55% reported that SUD care contributed to burnout. Over half (54%) had provided SUD care without feeling adequately trained, and 78% witnessed compromised care due to inadequate support, with attendings, APP, and residents reporting these more frequently than nurses. Similarly, 74% experienced distress when witnessing stigmatizing treatment of patients with SUD, with the highest rates in resident physicians (84%). Finally, 41% of participants reported that caring for patients with SUD was one of the most difficult parts of their job, with the highest rates among nurses (50%).

### Comfort with SUD care

Overall, most participants reported high levels of comfort with SUD diagnosis and treatment (Table 2). Comfort was highest for assessing alcohol withdrawal (89%) and opioid withdrawal (92%), with minimal variation across roles. Similarly, 88% felt comfortable diagnosing or recognizing SUD, 78% felt comfortable discussing medications for opioid use disorder (MOUD) with patients and initiating or administering methadone (70%) or buprenorphine (71%). Total 71% felt comfortable managing or addressing acute pain in patients with SUD. Fewer (49%) expressed comfort in discussing medications for alcohol use disorder (MAUD). Nurses reported high comfort with wound care for injection-related wounds (81%).

However, comfort was lower and more varied when it came to interventions for ongoing substance use and discharge planning. Comfort with counseling patients on overdose reversal with naloxone varied, with 81% of attendings feeling comfortable compared to 55% of nurses. Fewer respondents felt comfortable addressing in-hospital drug use (38%), counseling on safer alcohol use (35%) and drug use (41%), determining an appropriate level of care for SUD (43%) or connecting patients to outpatient SUD treatment (31%).

### Barriers to Addiction Care

The most frequently reported barrier to caring for patients with SUD were patient social barriers (84%) such as homelessness and transportation issues. Limited post-discharge resources were also a significant concern (68%), particularly for APP (86%) and attendings (81%). Access to expert consultation (56%) and harm reduction resources (53%) as barriers varied across roles, with physicians and APP reporting greater need. Similarly, attendings considered access to social work and peer support a barrier to care (69% and 50%, respectively), compared to nurses (28% and 33%, respectively). Patient interest in treatment as a barrier varied as well, from 79% among APP to 52% among nurses, 43% among residents and 36% among attendings. Knowledge about SUD treatment (32%), guidelines for managing OUD in the hospital (19%), and knowledge about pain management in SUD (19%) were less commonly perceived as barriers.

## Discussion

Our study aimed to characterize attitudes, comfort, and perceived barriers to SUD care among inpatient clinicians prior to the implementation of an ACS. While most participants recognized SUD as treatable and felt comfortable with diagnosis and acute management, significant gaps emerged in harm reduction strategies, discharge planning, and linkage to care. Limited social work and expert consultation were commonly cited as barriers. Notably, participants frequently reported moral distress and burnout related to caring for patients with SUD. These findings underscore the importance of assessing clinician perspectives prior to ACS implementation to tailor services to local needs, identify areas for support, and inform future qualitative research on interprofessional addiction care. They also mirror prior literature showing that hospital-based clinicians often feel unprepared to care for patients with SUD and experience distress and burnout in the absence of sufficient support.^6^^,^^11^^,^^12^ Our results reinforce these concerns and demonstrate that these challenges remain prevalent across diverse provider roles.

Clinicians reported higher comfort with SUD assessment and management, but lower confidence in postdischarge linkage and care for people continuing to use substances. Given that social determinants of health and patient interest in traditional treatment pathways were cited as barriers to effective care for PWUD, improving education and support for harm reduction and care linkage is essential. These trends are consistent with prior findings that identify postacute planning and harm reduction as persistent knowledge and practice gaps among inpatient teams.^4^^,^^7^^,^^10^^,^^13^ Training programs and health systems should emphasize strategies for overcoming these systemic challenges to enhance continuity of care.

Differences in attitudes, comfort, and perceived barriers across professional groups suggest that each discipline may require tailored support. While physicians and APPs frequently reported a lack of expert consultation and social work resources, nurses were significantly less likely to identifty these as barriera. Resident physicians were most likely to report distress related to stigmatizing care, and nurses most commonly reported that SUD care was one of the most difficult aspects of their job; both of these findingsmay indicate the challenges of frontline roles and the complex emotional labor involved. Nurses expressed more concern about enabling substance use than other provider groups, which may reflect discomfort or disagreement with harm reduction principles or approaches to withdrawal management. This concern may stem from opioid stewardship education, which emphasizes caution around opioid administration, potentially conflicting with evidence-based approaches to withdrawal care and harm reduction strategies. As with other findings in this study, this interpretation is based on self-reported data and may not reflect actual clinical behavior or institutional practices. Additionally, the delegation of opioid administration to nurses during withdrawal management may generate moral or emotional tension when interprofessional communication is limited and prescribers remain removed from the direct delivery of care. These dynamics highlight the need for qualitative research to explore role-specific experiences and inform targeted support for interdisciplinary teams.

High levels of clinician distress and burnout emerged as key findings, with over half of respondents feeling unsupported and many reporting witnessing stigmatizing treatment and compromised care due to inadequate SUD services. These results underscore the necessity of systems-level interventions that address the practical and emotional challenges of SUD care, including enhanced interdisciplinary collaboration, expanded access to harm reduction resources, and integrating ACS teams to provide real-time support. Our findings add to the literature by offering role-specific, preimplementation data from an interprofessional team, which can inform the structure and focus of future ACS efforts. At our institution, while the core structure of the ACS was shaped by available funding and staffing, this needs assessment helped guide how the team was deployed, and which areas of clinical focus and training were prioritized. While our findings are site-specific, they reflect widespread challenges in inpatient SUD care and suggest a replicable model for needs assessment to guide implementation. As more institutions consider adopting ACS models, this approach may serve as a practical framework for identifying gaps and aligning services with clinician needs.

An interesting finding was that a substantial proportion of nurses, APP, and resident physicians reported personal connections to individuals with SUD (38%, 36%, and 28%, respectively), compared to only 8% of attending physicians. Further research is needed to explore how these connections might influence attitudes, burnout, or care practices.

This study has several limitations, including single-site design and a lack of patient perspectives. Nonetheless, the findings provide valuable insights into the structural and educational needs for improving inpatient SUD care. As literature suggests ACS can improve clinician confidence, increase initiation of MOUD, and improve linkage to outpatient treatment, future work should assess whether ACS reduces the barriers identified in this study and explore additional strategies to enhance provider support and patient care outcomes.12., 13., 14.

## CRediT authorship contribution statement

**M. Holliday Davis:** Writing – review & editing, Writing – original draft, Project administration, Conceptualization. **Judy Chertok:** Writing – review & editing, Conceptualization. **Shoshana Aronowitz:** Writing – review & editing, Conceptualization. **Rachel French:** Writing – review & editing, Conceptualization. **Jeanmarie Perrone:** Writing – review & editing, Conceptualization. **Ashish P Thakrar:** Writing – review & editing, Conceptualization. **Samantha Huo:** Conceptualization. **Jacqueline Deanna Wilson:** Conceptualization. **Nia Bhadra-Heintz:** Writing – review & editing, Conceptualization. **Lilah Lesniak:** Project administration, Data curation. **Aidan Hecker:** Writing – review & editing. **Jessica Tolbert:** Project administration. **Margaret Lowenstein:** Writing – review & editing, Writing – original draft, Supervision, Methodology, Formal analysis, Data curation, Conceptualization.

## Declaration of competing interest

The authors declare that they have no known competing financial interests or personal relationships that could have appeared to influence the work reported in this paper.

## Appendix Supplementary materials

Image, application 1

Image, application 2
