# Supplementary material for: Caring for Hospitalized Patients with Substance Use Disorders: An Interprofessional Needs Assessment Survey
Source: Am J Med Open. 2025 Aug 20;14:100116. doi: 10.1016/j.ajmo.2025.100116 (PMC12481105; doi:10.1016/j.ajmo.2025.100116)
Supplement: Supplementary file 1 [file mmc1.pdf]

Addiction Consult Service Baseline Survey: Physicians/APPs

This survey is being conducted by a team of researchers at the University of Pennsylvania's Center for Addiction Medicine and Policy to better understand your experiences treating patients with substance use disorders, including opioid use disorder.

This survey is designed to assess your experiences caring for patients with substance use disorders, including opioid use disorder. We are interested in learning from frontline inpatient providers at Penn Presbyterian Medical Center ("Presby") in preparation for the implementation of an addiction medicine consult service this spring. You are being contacted because you are a physician or advanced practice provider who works at Presby.

The survey should take no more than 10 minutes to complete. The results will be confidential, and your responses will not be linked with your contact information. The results of this study will be used to inform future interventions aimed at improving the care for patients with substance use disorders.

At the end, there will be an optional area to provide your contact information to receive a $10 Amazon gift card.

Thank you for taking the time to share your experiences.

The following questions are about the frequency of caring for patients with substance use disorders in an inpatient setting at Penn Presbyterian Medical Center.

	Never	1-2 times	3-5 times	5-9 times	10 or more times
In a typical month, how often do you care for patients with alcohol use disorder? This could include patients presenting with complications related to their alcohol use (i.e. liver disease, alcohol withdrawal) or presentations for other medical issues in a patient with alcohol use disorder.	○	○	○	○	○
In a typical month, how often do you care for patients with opioid use disorder (OUD)? This could include patients presenting with complications related to their drug use (i.e. infections, withdrawal) or presentations for other medical issues in a patient with OUD.	○	○	○	○	○

In a typical month, how often do you care for patients with other substance use disorders (SUDs)? This could include patients presenting with complications related to their drug use or presentations for other medical issues in a patient who uses substances.	◯	◯	◯	◯	◯

The following questions ask about the frequency of practices for management of alcohol use disorder, opioid use disorder and other substance use in an inpatient setting at Penn Presbyterian Medical Center.

	Never	1-2 times	3-5 times	5-9 times	10 or more times
In a typical month, how often do you care for a patient with alcohol use disorder who was prescribed medication for alcohol use disorder (i.e. naltrexone or acamprosate) at discharge?	◯	◯	◯	◯	◯
In a typical month, how often do you care for a patient with opioid use disorder who was treated buprenorphine-naloxone (Suboxone) in the hospital?	◯	◯	◯	◯	◯
In a typical month, how often do you care for a patient with opioid use disorder who was treated methadone in the hospital?	◯	◯	◯	◯	◯
In a typical month, how often do you care for a patient with opioid use disorder receiving short-acting opioids (hydromorphone, oxycodone, etc) to manage acute pain and/or withdrawal?	◯	◯	◯	◯	◯
In a typical month, how often do you care for a patient who uses substances who was prescribed naloxone (or Narcan) for at discharge?	◯	◯	◯	◯	◯

In a typical month, how often do you (or someone on your team) counsel patients who use substances on harm reduction strategies (e.g. use of sterile supplies, fentanyl test strips, OD reversal, safer injection techniques)?	○	○	○	○	○

The next questions focus on your experiences caring for patients with substance use disorders.

Please rate your level of agreement with the following statements:

	Strongly disagree	Somewhat disagree	Neutral	Somewhat agree	Strongly agree
Patients with substance use disorders are more challenging to take care of compared to patients without addiction	○	○	○	○	○
Substance use disorders are treatable diseases	○	○	○	○	○
I do not have enough time to provide patients with substance use disorders the care they deserve	○	○	○	○	○
I worry about worsening or enabling addiction in my patients.	○	○	○	○	○
I feel manipulated by my patients who have substance use disorders	○	○	○	○	○
I feel unsupported in caring for patients with substance use disorders	○	○	○	○	○
There have been times when I have had to care for patients with substance use disorders whom I do not feel qualified to care for	○	○	○	○	○
Caring for patients with substance use disorders contributes to burnout for me	○	○	○	○	○
I have witnessed compromised patient care due to lack of resources or support for addiction treatment	○	○	○	○	○

I have felt distress when witnessing stigmatizing or unfair treatment provided by other members of the care team	◯	◯	◯	◯	◯
Caring for patients with substance use disorders is one of the most difficult parts of my job.	◯	◯	◯	◯	◯

The next questions focus on your level of preparation when caring for patients with substance use disorder.

Thinking about your education, training, and work experience, please describe how prepared you feel to:

	Very unprepared	Somewhat unprepared	Neutral	Somewhat prepared	Very prepared
Diagnose substance use disorders	◯	◯	◯	◯	◯
Assess for alcohol withdrawal	◯	◯	◯	◯	◯
Assess for opioid withdrawal	◯	◯	◯	◯	◯
Discuss medication options for alcohol use disorder (e.g. naltrexone, acamprosate)	◯	◯	◯	◯	◯
Discuss medication treatment for opioid use disorder (e.g.buprenorphine, methadone)	◯	◯	◯	◯	◯
Initiate methadone in the hospital	◯	◯	◯	◯	◯
Initiate buprenorphine in the hospital	◯	◯	◯	◯	◯
Manage acute pain in a patient with substance use disorder	◯	◯	◯	◯	◯
Determine the appropriate level of care for patients with substance use disorders (e.g. inpatient vs outpatient treatment)	◯	◯	◯	◯	◯
Connect patients from the hospital to outpatient substance use disorder treatment upon discharge	◯	◯	◯	◯	◯
Counsel a patient on safer ways to drink alcohol	◯	◯	◯	◯	◯
Counsel a patient about safer drug use practices (eg. the use of sterile syringes, safer injection practices, etc)	◯	◯	◯	◯	◯

Counsel a patient about overdose prevention with naloxone (Narcan)	◯	◯	◯	◯	◯
Address a patient's in-hospital illicit drug use	◯	◯	◯	◯	◯

Please indicate the degree to which each of the following is a barrier to providing comprehensive care to patients with substance use disorders, including opioid use disorders:

	Not at all a barrier	Neutral	Significant barrier
Knowledge about substance use disorder treatment	◯	◯	◯
Knowledge about pain management in patients with substance use disorders	◯	◯	◯
Access to clinical guidelines for managing opioid use disorder	◯	◯	◯
Access to harm reduction resources (sterile syringes, fentanyl test strips, naloxone)	◯	◯	◯
Access to expert clinical consultation for addiction care	◯	◯	◯
Availability of social work support for addiction care	◯	◯	◯
Availability of peer support for addiction care	◯	◯	◯
Availability of resources after discharge (e.g. inpatient/outpatient, skilled nursing facility placements)	◯	◯	◯
Patient social barriers (e.g. homelessness, transportation)	◯	◯	◯
Lack of patient interest in substance use treatment	◯	◯	◯
Other (please describe below):	◯	◯	◯

Other: __________________________________

Demographics

Please tell us about yourself:

Gender

◯ Male
◯ Female
◯ Non-binary
◯ Other

Age	◯ Under 30<br>◯ 30-39<br>◯ 40-49<br>◯ 50-59<br>◯ 60+
Ethnicity	◯ Hispanic or Latino<br>◯ Not Hispanic or Lation
Race	◯ Asian<br>◯ Black/African American<br>◯ Native American/American Indian<br>◯ Pacific Islander/Native Hawaiian<br>◯ White<br>◯ Other
Level of training	◯ Intern<br>◯ Resident<br>◯ Fellow<br>◯ Attending<br>◯ Nurse Practitioner/Physician Assistant
Years since graduating from Medical, NP or PA school	◯ 1-4<br>◯ 5-9<br>◯ 10-14<br>◯ 15+
Percent time spent in clinical care	◯ Less than 20%<br>◯ 20-50%<br>◯ 51-75%<br>◯ Greater than 75%
Please enter your preferred email address for your $10 Amazon gift card compensation for this survey	__________________________________
Do you have a close friend or family member with Substance Use Disorder? (optional)	◯ Yes<br>◯ No
Please feel free to share any information about your experiences. This information will be kept confidential and not linked with your personal information (optional)	__________________________________

Addiction Consult Service Baseline Survey: Nursing

This survey is being conducted by a team of researchers at the University of Pennsylvania's Center for Addiction Medicine and Policy to better understand your experiences treating patients with substance use disorders, including opioid use disorder.

This survey is designed to assess your experiences caring for patients with substance use disorders, including opioid use disorder. We are interested in learning from frontline inpatient providers at Penn Presbyterian Medical Center ("Presby") in preparation for the implementation of an addiction medicine consult service this spring. You are being contacted because you are a nurse who works at Presby.

The survey should take no more than 10 minutes to complete. The results will be confidential, and your responses will not be linked with your contact information. The results of this study will be used to inform future interventions aimed at improving the care for patients with substance use disorders, including opioid use disorder.

At the end, there will be an optional area to provide your contact information to receive a $10 Amazon gift card.

Thank you for taking the time to share your experiences.

The following questions are about the frequency of providing treatment for patients with substance use disorders in an inpatient setting at Penn Presbyterian Medical Center.

	Never	1-2 times	3-5 times	5-9 times	10 or more times
In a typical month, how often do you care for patients with alcohol use disorder? This could include patients presenting with complications related to their alcohol use (i.e. liver disease, alcohol withdrawal) or presentations for other medical issues in a patient with alcohol use disorder.	○	○	○	○	○
In a typical month, how often do you care for patients with opioid use disorder (OUD)? This could include patients presenting with complications related to their drug use (i.e. infections, withdrawal) or presentations for other medical issues in a patient with OUD.	○	○	○	○	○
In a typical month, how often do you care for patients with other substance use disorders (SUDs)? This could include patients presenting with complications related to their drug use or presentations for other medical issues in a patient who uses substances.	○	○	○	○	○

The following questions ask about the frequency of practices for management of alcohol use disorder, opioid use disorder and other substance use in an inpatient setting at Penn Presbyterian Medical Center.

	Never	1-2 times	3-5 times	5-9 times	10 or more times
In a typical month, how often have you cared for a patient with alcohol use disorder who was prescribed medication for alcohol use disorder (i.e. naltrexone or acamprosate) at discharge?	○	○	○	○	○
In a typical month, how often have you cared for a patient with opioid use disorder who was treated buprenorphine-naloxone (Suboxone) in the hospital?	○	○	○	○	○
In a typical month, how often have you cared for a patient with opioid use disorder who was treated methadone in the hospital?	○	○	○	○	○
In a typical month, how often have you cared for a patient with opioid use disorder receiving short-acting opioids (hydromorphone, oxycodone, etc) to manage acute pain and/or withdrawal?	○	○	○	○	○
In a typical month, how often have you cared for a patient who uses substances who was prescribed naloxone (or Narcan) for at discharge?	○	○	○	○	○
In a typical month, how often have you (or someone on your team) counseled patients who use substances on harm reduction strategies (e.g. use of sterile supplies, fentanyl test strips, OD reversal, safer injection techniques)?	○	○	○	○	○

The next questions focus on your experiences caring for patients with substance use disorders (SUDs).

Please rate your level of agreement with the following statements:

	Strongly disagree	Somewhat disagree	Neutral	Somewhat agree	Strongly agree
Patients with substance use disorders are more challenging to take care of compared to patients without addiction	○	○	○	○	○
Substance use disorders are treatable diseases	○	○	○	○	○
I do not have enough time to provide patients with substance use disorders the care they deserve	○	○	○	○	○
I worry about worsening or enabling addiction in my patients.	○	○	○	○	○
I feel manipulated by my patients who have substance use disorders.	○	○	○	○	○
I feel unsupported when I care for patients with substance use disorders	○	○	○	○	○
There have been times when I have had to care for patients with substance use disorders whom I do not feel qualified to care for	○	○	○	○	○
Caring for patients with substance use disorders contributes to burnout for me.	○	○	○	○	○
I have witnessed compromised patient care due to lack of resources or support for addiction treatment.	○	○	○	○	○
I have felt distress when witnessing stigmatizing or unfair treatment provided by other members of the care team.	○	○	○	○	○
Caring for patients with substance use disorders is one of the most difficult parts of my job.	○	○	○	○	○

The next questions focus on your level of preparation when caring for patients with substance use disorders.

Please describe how prepared you currently feel to do the following clinical activities:

	Very unprepared	Somewhat unprepared	Neutral	Somewhat prepared	Very prepared
Recognize substance use disorders	○	○	○	○	○
Assess for alcohol withdrawal	○	○	○	○	○
Asses for opioid withdrawal	○	○	○	○	○
Administer methadone in the hospital	○	○	○	○	○
Administer buprenorphine in the hospital	○	○	○	○	○
Address acute pain in a patient with a substance use disorder	○	○	○	○	○
Provide wound care for wounds related to injection drug use.	○	○	○	○	○
Counsel a patient on safer ways to drink alcohol	○	○	○	○	○
Counsel a patient about safer drug use practices (eg. use of sterile syringes, safer injection practices)	○	○	○	○	○
Counsel a patient about overdose prevention with naloxone (Narcan)	○	○	○	○	○
Address a patient's in-hospital illicit drug use	○	○	○	○	○

Please indicate the degree to which each of the following is a barrier to providing comprehensive care to patients with opioid use disorder and other substance use disorders:

		Significant barrier	Somewhat of a barrier	Not at all a barrier
1	Knowledge about substance use disorder treatment	○	○	○
	Knowledge about pain management in patients with substance use disorders	○	○	○
5	Access to clinical guidelines for managing substance use disorders	○	○	○
6				

	Access to harm reduction resources (sterile syringes, fentanyl test strips, naloxone)	◯	◯	◯
7	Access to expert clinical consultation for addiction care	◯	◯	◯
8	Availability of social work support for addiction care	◯	◯	◯
9	Availability of peer support for addiction care	◯	◯	◯
10	Availability of resources after discharge (e.g. inpatient/outpatient, skilled nursing facility placements)	◯	◯	◯
11	Patient social barriers (e.g. homelessness, transportation)	◯	◯	◯
12	Lack of patient interest in substance use treatment	◯	◯	◯
13	Other (please indicate below)	◯	◯	◯

If you chose other, please explain: ______________________________

Demographics

Please tell us about yourself:

Gender
- ◯ Male
- ◯ Female
- ◯ Non-binary
- ◯ Other

Age
- ◯ Under 30
- ◯ 30-39
- ◯ 40-49
- ◯ 50-59
- ◯ 60+

Ethnicity
- ◯ Hispanic or Latino
- ◯ Not Hispanic or Lation

Race
- ☐ Asian
- ☐ Black/African American
- ☐ Native American/American Indian
- ☐ Pacific Islander/Native Hawaiian
- ☐ White
- ☐ Other

Level of training
- ◯ Certified Nursing Assistant
- ◯ Licensed Nursing Assistant
- ◯ Registered Nurse, Associates Degree
- ◯ Registered Nurse, Bachelors Degree

Years since graduating from Nursing school?	◯ 1-4<br>◯ 5-9<br>◯ 10-14<br>◯ 15+
Percent time spent in clinical care	◯ Less than 20%<br>◯ 20-50%<br>◯ 51-75%<br>◯ Greater than 75%
Please enter your preferred email address for your $10 Amazon gift card compensation for this survey	______________________________
Do you have a close friend or family member with Substance Use Disorder? (optional)	◯ Yes<br>◯ No
Please feel free to share any information about your experiences. This information will be kept confidential and not linked with your personal information (optional)	______________________________
